# Sex-related differences in lipid peroxidation and photoprotection in *Pistacia lentiscus*

**DOI:** 10.1093/jxb/ert446

**Published:** 2013-12-30

**Authors:** Marta Juvany, Maren Müller, Marta Pintó-Marijuan, Sergi Munné-Bosch

**Affiliations:** Departament de Biologia Vegetal, Facultat de Biologia, Universitat de Barcelona, Avinguda Diagonal, 643, E-08028 Barcelona, Spain

**Keywords:** Dioecy, mastic tree (*Pistacia lentiscus*), oxidative stress, photoprotection, reproductive effort, sexual dimorphism.

## Abstract

Sex-related differences in the response of dioecious plants to abiotic stress have been poorly studied to date. This work explored to what extent sex may affect plant stress responses in *Pistacia lentiscus* L. (Anacardiaceae), a tree well adapted to Mediterranean climatic conditions. It was hypothesized that a greater reproductive effort in females may increase oxidative stress in leaves, particularly when plants are exposed to abiotic stress. Measurements of oxidative stress markers throughout the year revealed increased lipid peroxidation in females, but only during the winter. Enhanced lipid peroxidation in females was associated with reduced photoprotection, as indicated by reduced tocopherol levels and nonphotochemical quenching (NPQ) of chlorophyll fluorescence. Enhanced lipid peroxidation in females was also observed at predawn, which was associated with increased lipoxygenase activity and reduced cytokinin levels. An analysis of the differences between reproductive (R) and nonreproductive (NR) shoots showed an enhanced photoprotective capacity in R shoots compared to NR shoots in females. This capacity was characterized by an increased NPQ and a better antioxidant protection (increased carotenoid and tocopherol levels per unit of chlorophyll) in R compared to NR shoots. It is concluded that (i) females exhibit higher lipid peroxidation in leaves than males, but only during the winter (when sex-related differences in reproductive effort are the highest), (ii) this is associated with a lower photoprotective capacity at midday, as well as enhanced lipoxygenase activity and reduced cytokinin levels at predawn, and (iii) photoprotection capacity is higher in R relative to NR shoots in females.

## Introduction

Studies on dimorphism between sexes in dioecious plants have not only shown differential reproductive traits in males and females but also significant differences in vegetative traits generally associated with a greater reproductive effort in females (Barrett and Hough, 2013). There is evidence that females present a higher investment of nutrients in reproduction at the expense of vegetative growth, and therefore females show lower productivity than males (Gross and Soule, 1981; Ågren, 1988; Korpelainen, 1992; Cipollini and Whigham, 1994; Zluvova *et al.*, 2010). Consequently, environmental stress, caused by less-than-optimal light, nutrition, or water conditions, often favours maleness (reviewed by Korpelainen, 1999). Nevertheless, several exceptions exist, particularly in the case of herbaceous perennials (reviewed by Obeso, 2002). Therefore, more studies are needed to better understand the consequences of sex dimorphism in the adaptation of plants to environmental stresses. This information can provide helpful insights into better understanding the biology of dioecious plants and help to improve management practices in their natural habitat.

Reproductive effort implies that both sexes differ in a range of morphological, physiological, and life-history traits. Some studies in trees have tested the gender-specific response against a number of environmental stresses such as drought (Li *et al.*, 2004; Varga and Kytoviita, 2008; Xu *et al.*, 2008; Rozas *et al.*, 2009; Chen *et al.*, 2010), low temperatures (Zhang *et al.*, 2011), salinity (Chen *et al.*, 2010), atmospheric CO_2_ enrichment (Wang and Curtis, 2001), enhanced UV-B radiation (Xu *et al.*, 2010), nutrient deficiency (Montesinos *et al.*, 2012), excess manganese, and a combination of different stresses (Chen *et al.*, 2010; Zhang *et al.*, 2010). Although these studies conclude that in front of environmental stresses females seem to be more sensitive and usually experience greater negative effects, other studies in perennial herbs indicate that females are equally or even more resistant than males to environmental stresses, such as drought (Oñate and Munné-Bosch, 2009; Morales *et al.*, 2013). Due to the very limited amount of studies, it is still not clear whether such differential effects of sexual dimorphism on plant stress responses are species-specific or caused by specific ecological circumstances; therefore, further research in other species and habitats is needed to strengthen knowledge on this topic.

In Mediterranean-type ecosystems plants are exposed to marked seasonal variations throughout the year, which are usually characterized by a summer drought followed by a rainy autumn and lower temperatures during the winter. Even though sex-related differences in the response of dioecious Mediterranean plants to photo-oxidative stress have not been examined thus far, other studies have shown that dioecious plants differ in the physiological response to drought between sexes. Females of *Populus yunnanensis* growing in China exhibited more growth inhibition, gas exchange rate depression, reactive oxygen species (ROS) accumulation, and more damage to cell organelles than did males under drought (Chen *et al.*, 2010). Female individuals of *Populus cathayana* growing in China were also more responsive and showed greater negative effects (in terms of growth, net photosynthesis, and oxidative stress) than did males when grown under environments with increased drought stress and elevated temperature (Xu *et al.*, 2008). Therefore, this work hypothesized that females of *Pistacia lentiscus* L., a tree widely distributed along the Mediterranean basin, would also exhibit higher photo-oxidative stress than males, particularly during the summer. Indeed, previous studies already showed evidence for a higher sensitivity to drought stress in terms of leaf gas exchange in females (Correia *et al.*, 1992; Jonasson *et al.*, 1997; Barradas and Correia, 1999), but no studies have investigated sex-related differences in photo-oxidative stress in this or other Mediterranean dioecious plants. These studies can undoubtedly bring new insights into better understanding the biochemical mechanisms behind stress tolerance and provide clues to better understand sex biases in nature. Furthermore, to better understand photoprotection and photo-oxidative stress in fruit-bearing plants, this work specifically examined the effects of the reproductive effort in females by (i) analysing the differences between reproductive (R) and nonreproductive (NR) shoots, and (ii) conducting manipulative experiments by detaching fruits on R shoots.

## Materials and methods

### Plant material, growth conditions, and samplings

This study was conducted using *Pistacia lentiscus* L. (Anacardiaceae), a dioecious evergreen tree, typical of Mediterranean dry and semiarid climates. Fifty-five plants with a height between 40 and 110cm were purchased in Bioriza (Cornella de Terri, Girona, Spain) in the spring of 2009 and were homogeneously transplanted in an area of 30 m^2^ to the experimental fields of the Faculty of Biology at the University of Barcelona (Barcelona, Spain, 41° 22′ 59″ N 02° 06′ 44″ E, 60 m above sea level). Plants were grown under Mediterranean climatic conditions and received water exclusively from rainfall before and during the study. During March 2012, at flowering time, a group of 12 plants (six females and six males) were selected and tagged for experiments. Two different experiments were performed with these plants.

In the first experiment, leaves of both sexes were sampled at midday during spring (28 March), summer (28 July), autumn (29 October), and winter (24 January) of 2012–2013 to evaluate sex-related differences in seasonal variations of photo-oxidative stress markers. In males, inflorescences appeared during March and abscised during May, while in females inflorescences appeared in March and fruits developed from June to February. In this experiment, leaves from R and NR shoots were not differentiated.

In the second experiment, leaves from NR shoots of both males and females were sampled at midday at regular intervals between 29 October 2012 and 24 January 2013. During the same period, leaves from both R and NR shoots of females were used to specifically evaluate the effects of the reproductive effort in females during winter. Furthermore, a manipulative experiment on females was conducted during the same period by removing fruits at the start of the experiment in R shoots (without fruit) and its response was compared to leaves of R shoots (with fruit). Finally, to determine the possible diurnal variations in photo-oxidative stress markers during winter, all leaf types mentioned before were collected at predawn (1h before sunrise), midday (at maximum diurnal solar radiation), and evening (3h after sunset) during 25 January 2013. In this experiment, lipoxygenase activity and hormone levels (cytokinins, abscisic acid (ABA), and jasmonic acid (JA)) were additionally measured to better understand the causes of the sex-related differences in lipid peroxidation at predawn and midday.

Fully developed, young leaves were always selected for measurements. For biochemical analyses, samples were immediately frozen in liquid nitrogen and stored at –80 °C until measurements.

### Climatological conditions and leaf water contents

Climatological conditions during experiments were monitored every 5min by means of a weather station situated at 100 m from the experimental fields. Data was obtained by a photosynthetically active photon flux density (PPFD) pyranometer sensor CM11 (KIPP and ZONEN, Delft, The Netherlands) and a HMP35AC thermohygrometer (Vaisala, Helsinki, Finland). Leaf water status was estimated by measuring the relative water content (RWC) as 100 × (FW – DW)/(TW – DW), where FW is the fresh weight, TW is the weight of the leaves rehydrated for 24h at 4 °C in darkness, and DW corresponds to the dry weight obtained after overdrying the samples for 24h at 80 °C.

### Extent of lipid peroxidation

The extent of lipid peroxidation was estimated spectrophotometrically by the amount of malondialdehyde (MDA) in leaves, following the method described by Hodges *et al.* (1999), which takes into account the possible influence of interfering compounds in the thiobarbituric acid-reactive substances assay.

### Photoinhibition and photoprotection

The maximum efficiency of PSII photochemistry (*F*_v_/*F*_m_), which is indicative of photoinhibition to PSII, was measured with a pulse-modulated fluorimeter Imaging PAM (Walz, Effeltrich, Germany) according to van Kooten and Snel (1990). Measurements were performed after 2h of dark adaptation.

For pigment (chlorophyll (Chl) a+b, carotenoids, and anthocyanins) analysis, samples were ground in liquid nitrogen and repeatedly extracted with ice-cold 100% methanol (v/v) using sonication until the extract was colourless. Chl and carotenoid contents were estimated spectrophotometrically. Specific absorption coefficients reported by Lichtenthaler (1987) were used. Anthocyanin content was determined with the acidification of the same extract with HCl and measured spectrophotometrically as described by Gitelson *et al.* (2001). Carotenoid and anthocyanin levels were given both on a dry weight and Chl basis, the latter indicating the capacity of photoprotection per unit of Chl.

For tocopherol analyses, samples were ground in liquid nitrogen and repeatedly extracted with ice-cold 100% methanol (v/v) using sonication until the extract was colourless. Τocopherols (including all four homologues) were determined by HPLC as described by Amaral *et al.* (2005). Since differences in α-tocopherol levels were found between sexes, this work further evaluated the oxidation state of α-tocopherol in the diurnal cycle by measuring α-tocopherol quinone (α-TQ). For α-TQ analyses, samples were extracted as described for tocopherols, but HPLC analyses were performed as described by Munné-Bosch and Alegre (2003). All tocopherol homologues and α-TQ were identified by coelution with authentic standards (Sigma, Steinheim, Germany), and identification of α-TQ was further confirmed by matching UV/visible spectra. Tocopherol levels were given both on a dry weight and Chl basis, the latter indicating the capacity of photoprotection per unit of Chl. α-TQ measurements allowed this work to additionally calculate the oxidation state of α-tocopherol, given as α-TQ/α-Tt, where α-Tt is the sum of α-tocopherol plus α-TQ.

Leaf gas exchange and Chl fluorescence measurements were additionally performed at the end of the experiment to examine light intensity-dependent curves of photosynthesis and photoprotection in terms of excess energy dissipation as heat (NPQ). All measurements were perfomed between 24 and 26 January 2013. Leaf gas exchange measurements coupled to Chl fluorescence were performed on attached leaves with a portable infrared gas analyser (LI-6400 system, LI-COR, Lincoln, NE, USA) with a leaf chamber fluorometer. To mimic environmental conditions at midday, leaves were maintained at 15 °C and the supplied CO_2_ concentration inside the cuvette was set at 400 μmol mol^−1^ during measurements. Measurements started after acclimating leaves at the highest light intensity (2500 μmol quanta m^–2^ s^–1^), followed by measurements at decreasing light intensities at intervals of 500 μmol quanta m^–2^ s^–1^ until final acclimation to darkness. Net CO_2_ assimilation (*A*) and stomatal conductance (*g*_s_) rates were calculated on steady state photosynthesis as described by von Caemmerer and Farquhar (1981). Chl fluorescence was determined concomitantly at each point of gas exchange measurement. From the fluorescence measurements, the relative efficiency of the photosystem II (PSII) photochemistry (Φ_PSII_) and NPQ (= (*F*_m_ – *F*_m_′)/*F*_m_′) were determined according to van Kooten and Snel (1990). The instantaneous water use efficiency (WUE) was calculated as *A*/*g*_s_, where *A* is the net CO_2_ assimilation rate and *g*_s_ is the stomatal conductance.

### Lipoxygenase activity and hormone levels

To further evaluate the causes of increased lipid peroxidation in females at predawn, which occurred irrespective of sex-related differences in photoprotection, this work examined lipoxygenase activity and hormone levels. Hormone analyses included determination of cytokinins as an indicator of sink strength and ABA and JA, which are known regulators of lipoxygenase activity (Melan *et al.*, 1993; Fischer *et al.*, 1999).

Lipoxygenase activity was assayed as described by Fukuchi-Mizutani *et al.* (2000). In short, samples were extracted for 30min with 0.01M potassium phosphate buffer, pH 7.2, using ultrasonication. After centrifugation, the pellet was re-extracted with the same solvent. Supernatants were pooled and an aliquot was added to the reaction mixture (1ml) at 37 °C. The reaction mixture consisted of 0.1M sodium acetate buffer, pH 5, 0.0025% Tween 20, and 0.2mM linoleic acid and activity was estimated spectrophotometrically at 234nm.

The levels of cytokinins, including zeatin, zeatin riboside, isopentenyladenosine (IPA) and 2-isopentenyladenine (2-iP), ABA, and JA, were determined by UPLC-MS/MS as described by Müller and Munné-Bosch (2011). Internal standards were used for estimating recovery rates for quantification.

### Statistical analyses

In the first experiment, statistical differences between time through the year and sex were tested by two-way factorial analysis of variance (ANOVA), using time and sex as factors. The second experiment assessed (1) the sex effect, comparing NR shoots from both males and females; (2) the shoot effect, comparing R and NR shoots in females; and finally (3) the fruit effect, comparing R shoots with and without fruits. Fruits were removed in the latter. Statistical differences between time and sex, shoot, or fruit were tested by two-way factorial analysis of variance (ANOVA), using time and sex, shoot, or fruit as factors. Additionally, Student’s t-tests were used to specifically evaluate differences between sexes at a given time point. In all cases, differences were considered significant at a probability level of *P* < 0.05.

## Results

### Sex-related seasonal variations in lipid peroxidation and photoprotection

Climatological conditions during the experiment were typical of the Mediterranean climate, with a summer drought (total rainfall between June and August was below 60mm) followed by a rainy autumn (more than 100mm during October) and lower temperatures during winter (below 10 °C at midday, Supplementary Fig. S1, available at *JXB* online). During samplings, midday temperatures and PPFDs ranged between 10 °C and 32 °C, and between 876 and 1940 µmol m^–2^ s^–1^ (during winter and summer, respectively, Supplementary Table S1). Despite severe summer drought, both males and females plants maintained RWC values above 75% and *F*_v_/*F*_m_ above 0.80 throughout the experiment (Supplementary Fig. S2). Chl a+b contents were however strongly reduced during spring and summer, although no sex-related differences were observed. Neither seasonal nor sex-related differences were observed in lipid peroxidation, estimated as MDA levels, although females showed higher levels than males both during autumn (38%) and winter (46%, *P* < 0.05, Student’s t-test, Supplementary Fig. S2). Total anthocyanin, carotenoid and α-tocopherol levels, either per dry weight or per Chl unit, showed seasonal variations but no sex-related differences. However, anthocyanin and α-tocopherol levels per unit of Chl were 25% lower in females than in males during winter (Supplementary Fig. S3).

### Sex-related differences in lipid peroxidation and photoprotection during winter

**Fig. 1.**
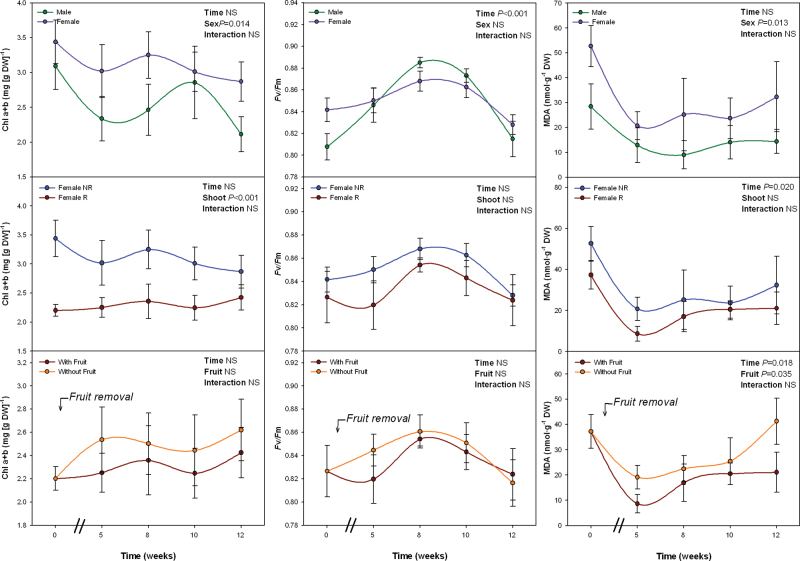
Sex-related differences during winter in the maximum efficiency of PSII photochemistry (*F*_v_/*F*_m_), and levels of chlorophyll (Chl) a+b and malondialdehyde (MDA) in *Pistacia lentiscus*. Data represent mean ± SE of six individuals. Significant differences between groups were tested by two-way factorial analyses of variance (ANOVA) with time and plant sex (females vs. males), shoot (R, reproductive; NR, nonreproductive), or fruit (shoots with and without fruits) as factors. NS, not significant (this figure is available in colour at *JXB* online).

To better understand such sex-related differences in lipid peroxidation and photoprotection during winter a second experiment was conducted. Sex-, shoot- and fruit-related differences were tested. First, NR shoots of both males and females were selected to evaluate sex-related differences. Fruits were developing in R shoots of females during samplings, while inflorescences had abscised several months ago in males; therefore, although sampled shoots had not reproduced, gender-related differences in reproductive effort were maximal at the whole-plant level during winter. Second, R and NR shoots of females were compared to evaluate shoot-related differences in females. Third, female R shoots with and without fruits (in which fruits were manually eliminated at the beginning of the experiment) were compared to evaluate effects specifically caused by fruit development. During samplings, plants were exposed to suboptimal low temperatures (ranging between 10 °C and 15.3 °C at midday, Table 1). RWC values kept always above 80% in all cases (Supplementary Fig. S4). The *F*_v_/*F*_m_ did not differ between males and females, but Chl levels and MDA levels were significantly higher in females than in males (Fig. 1). However, neither *F*_v_/*F*_m_ nor MDA levels differed between R and NR of females, while Chl levels were lower in the former. Fruit removal in female R shoots did not alter Chl levels or *F*_v_/*F*_m_, but increased MDA levels. R shoots without fruits behaved as NR shoots in terms of MDA accumulation (Fig. 1).

**Table 1.** Climatological conditions during the measurement days of experiment 2Data correspond to measurements performed at maximum diurnal incident PPFD (at midday). PPFD, maximum photosynthetically active photon flux density; T_air_, air temperature; RH, relative humidity.

Day	Time (weeks)	PPFD (μmol m^–2^ s^–1^)	Tair (°C)	RH (%)
29.10.2012	0	1152	14.6	35
04.12.2012	5	776	14.9	46
20.12.2012	8	290	15.1	57
08.01.2013	10	760	15.3	53
24.01.2013	12	876	10.0	52

**Fig. 2.**
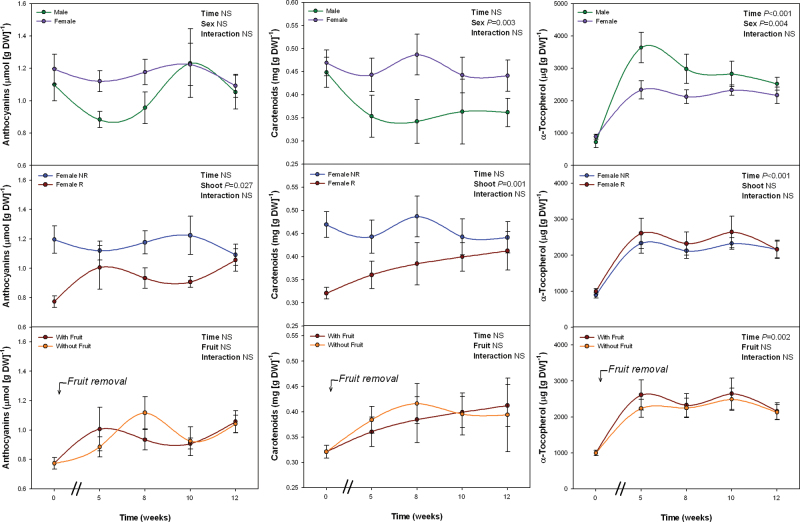
Sex-related differences during winter in the levels of total anthocyanins (Ant), carotenoids (Car) and α-tocopherol in *Pistacia lentiscus*. Data represent mean ± SE of six individuals. Significant differences between groups were tested by two-way factorial analyses of variance (ANOVA) with time and plant sex (females vs. males), shoot (R, reproductive; NR, nonreproductive), or fruit (shoots with and without fruits) as factors. NS, not significant (this figure is available in colour at *JXB* online).

Sex-related differences in α-tocopherol levels followed exactly the opposite trend than MDA levels. α-Tocopherol levels were lower in females than in males, both on a dry weight (Fig. 2) and on a Chl basis (Supplementary Fig. S5). Anthocyanin did not differ between males and females, but carotenoid levels were higher in the latter (Fig. 2). However, neither anthocyanin nor carotenoid levels per Chl unit differed between sexes (Supplementary Fig. S5). Anthocyanin levels were lower in R than in NR shoots of females (Fig. 2), but differences disappeared when expressed on a Chl basis (Supplementary Fig. S5). Female R shoots not only showed lower Chl levels than NR shoots (Fig. 1), but also higher carotenoid and α-tocopherol levels per unit of Chl (Supplementary Fig. S5). Fruit removal did not cause any effect on anthocyanin, carotenoid, or tocopherol levels (Fig. 2 and Supplementary Fig. S5).

**Fig. 3.**
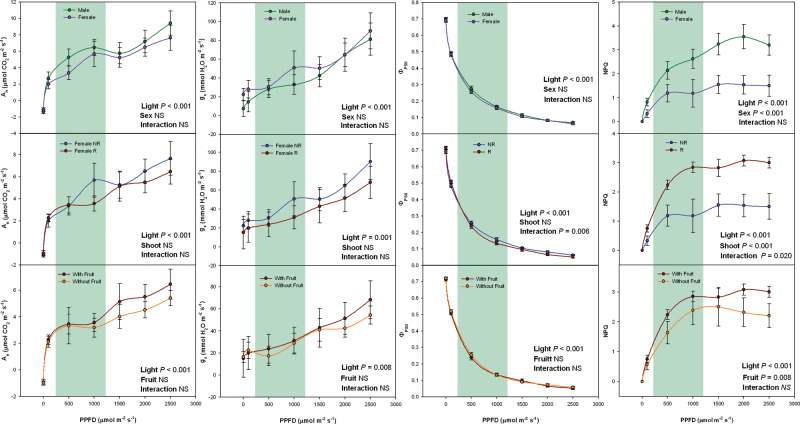
Sex-related differences during winter in the CO_2_ assimilation rate (*A*), stomatal conductance (*g*_s_), photochemical PSII efficiency (Φ_PSII_), and nonphotochemical quenching (NPQ) response curves to photosynthetically active photon flux density (PPFD) in *Pistacia lentiscus*. Data represent mean ± SE of six individuals. Significant differences between groups were tested by two-way factorial analyses of variance (ANOVA) with time and plant sex (females vs. males), shoot (R, reproductive; NR, nonreproductive), or fruit (shoots with and without fruits) as factors. NS, not significant. Green shading indicates midday sampling photosynthetically active photon flux density range during winter (this figure is available in colour at *JXB* online).

Light intensity-dependent curves of net CO_2_ assimilation (*A*) and stomatal conductance (*g*_s_) rates, as well as the quantum yield of PSII photochemistry (Φ_PSII_) and NPQ were additionally measured during winter (Fig. 3). While Φ_PSII_, *A*, and *g*_s_ did not differ between sexes, NPQ values indicated lower dissipation of excess energy by heat in females than in males. Furthermore, NPQ was much (up to 2-fold) higher in R than in NR of females, and fruit removal reduced the extent of NPQ, particularly at the highest light intensities (Fig. 3).

### Diurnal sex-related differences in lipid peroxidation and possible causes

**Fig. 4.**
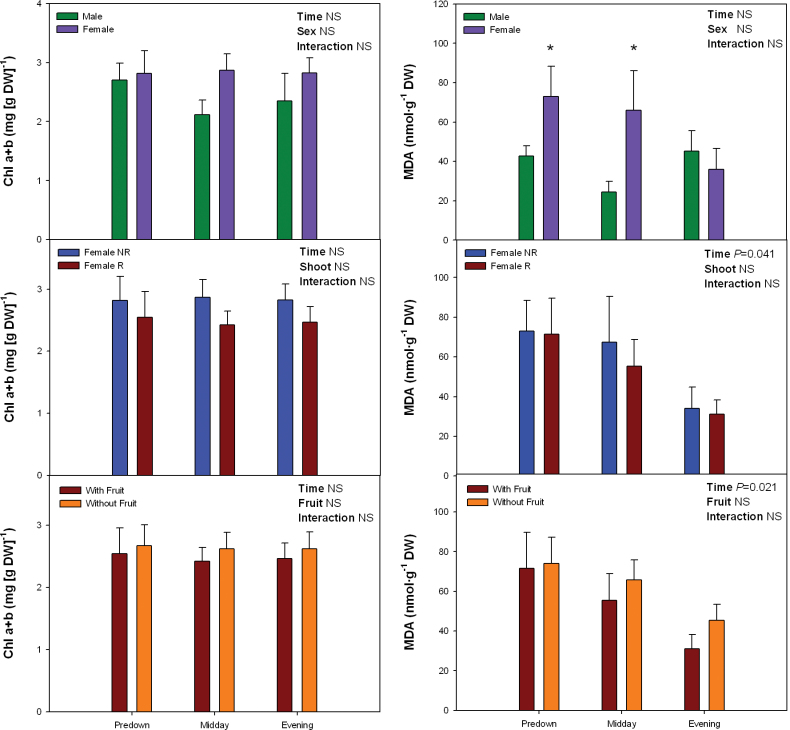
Sex-related differences in the diurnal variations in levels of chlorophyll (Chl) a+b and malondialdehyde (MDA) in *Pistacia lentiscus* during winter (25 January). Data represent mean ± SE of six individuals. Significant differences between groups were tested by two-way factorial analyses of variance (ANOVA) with time and plant sex (females vs. males), shoot (R, reproductive; NR, nonreproductive), or fruit (shoots with and without fruits) as factors. Asterisks indicate significant differences between males and females, R and NR shoots, or shoots with and without fruits at a given time point (Student’s t-test, *P* < 0.05). NS, not significant (this figure is available in colour at *JXB* online).

The extent of lipid peroxidation, together with the pigment and tocopherol levels (including its oxidation state), were examined on a diurnal basis during winter. Chl levels were not altered by daytime, sex, shoot, or fruit removal (Fig. 4). MDA levels were neither affected by any of these factors, except for time of day in females. The extent of lipid peroxidation decreased both in R and NR of females during the afternoon, while this diurnal pattern was not observed in males. In other words, females showed higher MDA levels than males at predawn and midday (*P* < 0.05, Student’s t-test), but not during the evening (Fig. 4), which is indeed consistent with the differences observed during winter at midday (Fig. 1).

**Fig. 5.**
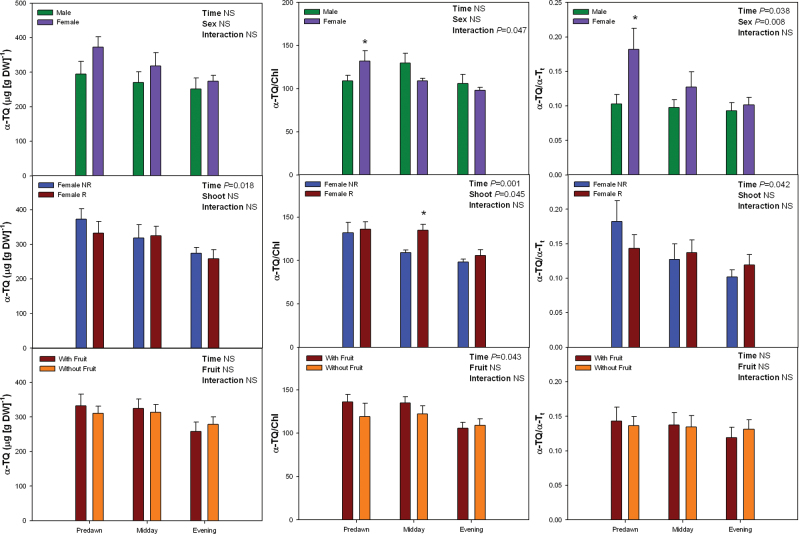
Sex-related differences in the diurnal variations in levels of α-tocopherol quinone (α-TQ), given both per g of dry weight (DW) and per unit of chlorophyll (Chl) a+b, and the oxidation state of α-tocopherol (estimated as α-TQ/α-T_t_, where α-T_t_ = α-T + α-TQ) in *Pistacia lentiscus* during winter (25 January) (this figure is available in colour at *JXB* online). Data represent mean ± SE of six individuals. Significant differences between groups were tested by two-way factorial analyses of variance (ANOVA) with time and plant sex (females vs. males), shoot (R, reproductive; NR, nonreproductive), or fruit (shoots with and without fruits) as factors. Asterisks indicate significant differences between males and females, R and NR shoots, or shoots with and without fruits at a given time point (Student’s t-test, *P* < 0.05). NS, not significant (this figure is available in colour at *JXB* online).

Such diurnal variations occurred in parallel with sex-related differences in the oxidation state of α-tocopherol (Fig. 5), but not with levels of anthocyanins, carotenoids, or α-tocopherol neither on a DW nor on a Chl basis (Supplementary Figs. S5 and S6). Reductions of MDA levels during the day coincided with the decrease of α-TQ levels on a DW basis and the oxidation state of α-tocopherol, given as α-TQ/α-Tt, where α-Tt is total α-tocopherol (reduced plus oxidized, Fig. 5).

**Fig. 6.**
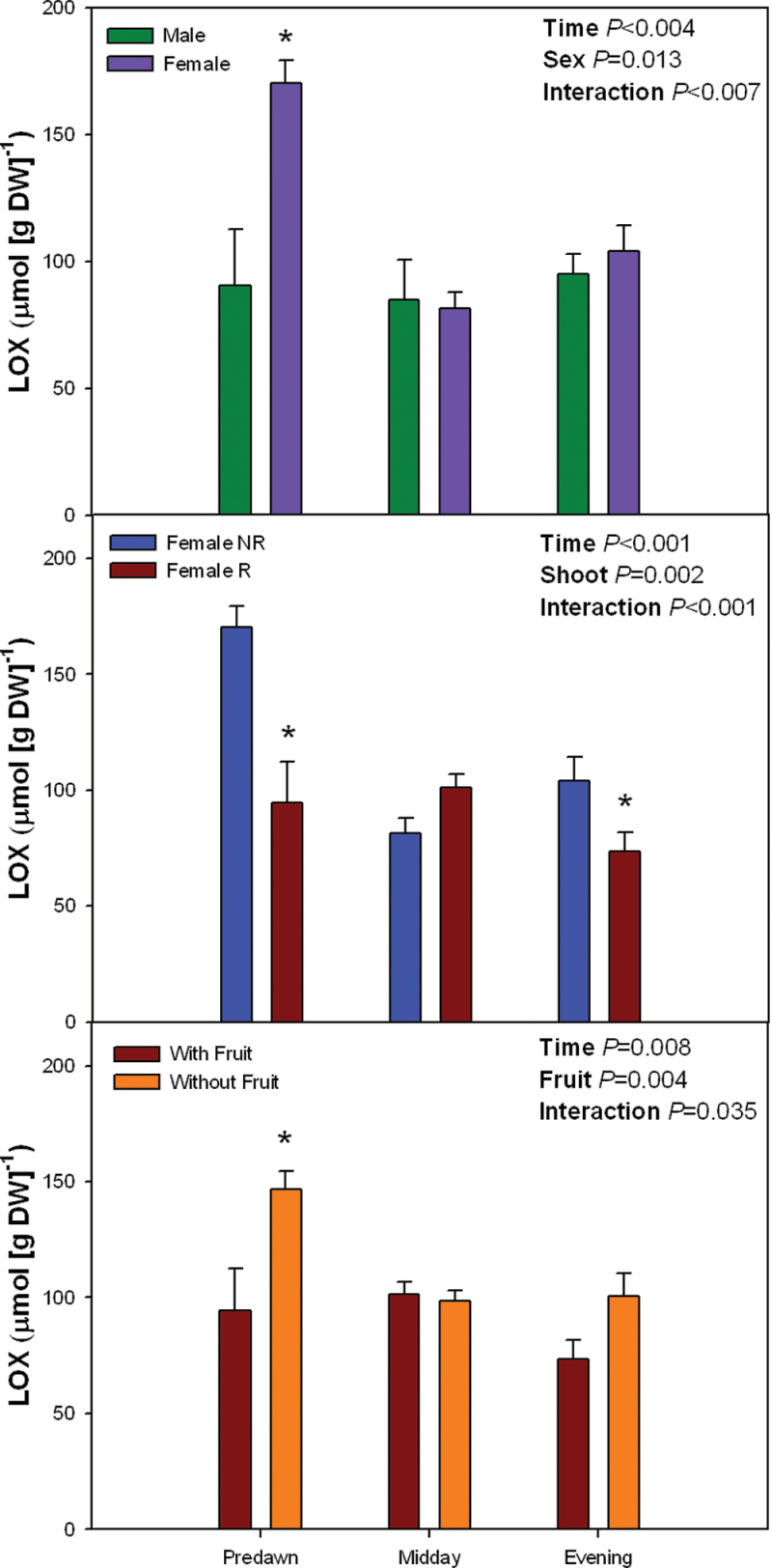
Sex-related differences in the diurnal variations in lipoxygenase (LOX) activity in *Pistacia lentiscus* during winter (25 January). Data represent mean ± SE of six individuals. Significant differences between groups were tested by two-way factorial analyses of variance (ANOVA) with time and plant sex (females vs. males), shoot (R, reproductive; NR, nonreproductive), or fruit (shoots with and without fruits) as factors. Asterisks indicate significant differences between males and females, R and NR shoots, or shoots with and without fruits at a given time point (Student’s t-test, *P* < 0.05). NS, not significant (this figure is available in colour at *JXB* online).

Furthermore, higher MDA levels and oxidation state of α-tocopherol in females than in males at predawn also coincided with increased lipoxygenase activity in the former (Fig. 6). Lipoxygenase activity was 85% higher in females than in males during predawn (*P* < 0.05, Student’s t-test), but values did not differ between sexes at midday or evening. Similar diurnal differences were observed between R and NR shoots in females, and fruit removal increased lipoxygenase activity in R shoots at predawn (and to a lower extent at evening, *P* < 0.05, Student’s t-test,), but not at midday (Fig. 6).

**Fig. 7.**
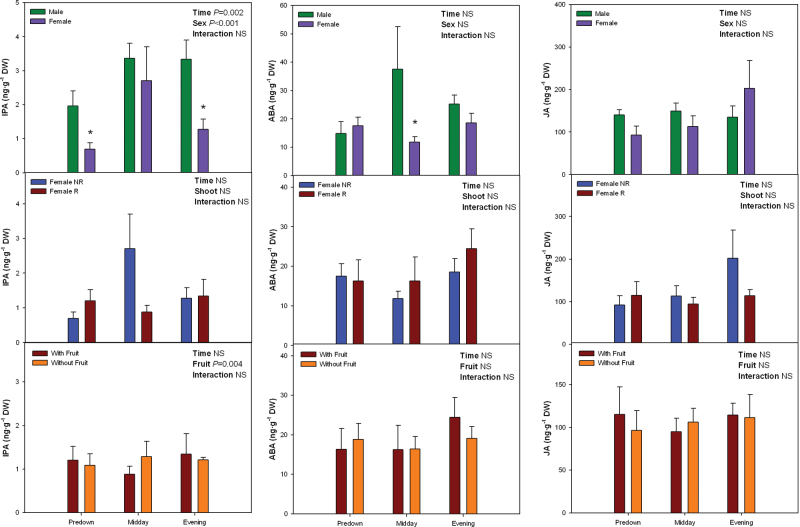
Sex-related differences in the diurnal variations in isopentenyladenosine (IPA), abscisic acid (ABA) and jasmonic acid (JA) levels in *Pistacia lentiscus* during winter (25 January). Data represent mean ± SE of six individuals. Significant differences between groups were tested by two-way factorial analyses of variance (ANOVA) with time and plant sex (females vs. males), shoot (R, reproductive; NR, nonreproductive), or fruit (shoots with and without fruits) as factors. Asterisks indicate significant differences between males and females, R and NR shoots, or shoots with and without fruits at a given time point (Student’s t-test, *P* < 0.05). NS, not significant (this figure is available in colour at *JXB* online).

Diurnal variations in hormonal levels were determined in males and females to further examine the sex-related differences in the extent of lipid peroxidation at predawn, which could not be related to sex-related differences in photoprotection. Increased MDA levels in females compared to males at predawn (Fig. 6) were associated with a 75% reduction of IPA levels at predawn in the former (*P* < 0.05, Student’s t-test, Fig. 7). Zeatin riboside levels were instead lower in females than in males at midday (*P* < 0.05, Student’s t-test), and 2-iP levels were lower in females than in males at midday and evening (*P* < 0.05, Student’s t-test, Supplementary Fig. S8). Neither ABA nor JA differed between sexes at predawn (Fig. 7). In contrast, ABA levels were reduced by 72% in females compared to males at midday (*P* < 0.05, Student’s t-test, Fig. 7), which was associated with a reduction by 20–40% in the WUE at and above 1500 μmol quanta m^–2^ s^–1^ in females compared to males (*P* < 0.05, Student’s t-test, Supplementary Fig. S9).

## Discussion

In Mediterranean-type ecosystems, dioecious plants are representative of ecologically relevant species. Both in coastal and more dry, continental areas, dominant trees, such as the dioecious *Pistacia lentiscus* and *Juniperus thurifera*, are typically found at (or close) the seashore and the mountain regions, respectively, of the western Mediterranean basin. Despite the dominant role of these plant species in their respective habitats, little is known about gender-specific effects on their physiology, except some studies showing higher drought sensitivity for females in both species in terms of gas exchange and growth (Correia *et al.*, 1992; Jonasson *et al.*, 1997; Barradas and Correia, 1999; Rozas *et al.*, 2009). These studies show that females are more sensitive than males to drought stress in terms of productivity and leaf gas exchange, females of *Pistacia lentiscus* showing reduced CO_2_ assimilation rates and PSII efficiency compared to males. The present study hypothesized that this reduced PSII efficiency may be associated with an increased photo-oxidative stress in females. Interestingly, increased lipid peroxidation associated with lower photoprotection was observed in females, but only during the winter. Seasonal variations in lipid peroxidation did not reveal sex-related differences during spring or summer, when plants are exposed to excess light energy, but during winter only. During the winter, plants were exposed to suboptimal temperatures (between 10 °C and 15.3 °C at midday), and the sex-related differences in reproductive effort were the highest, females developing fruits while males not reproducing. This is in agreement with the fact that in dioecious species the cost of reproduction involves the prioritization of resources in fruit development rather than in vegetative growth or protection in females. This major investment in reproduction has been generally associated with a disadvantage in terms of leaf gas exchange and productivity, leading even in some cases to cause increased oxidative stress and cellular injuries, particularly under adverse conditions (Xu *et al.*, 2008; Chen *et al.*, 2010; Zhang *et al*., 2010, 2011). The current work additionally shows that this increased sensitivity of females to lipid peroxidation is particularly observed in NR shoots. Indeed, when females R and NR shoots were compared, R shoots resulted to be more photoprotected, not only by an enhanced excess energy dissipation by heat (as indicated by a higher NPQ), but also by an improved antioxidant protection (as indicated by higher carotenoid and tocopherol levels per Chl unit).

Photoprotection capacity differed between male and female plants. Despite PSII efficiency and CO_2_ assimilation rates were identical between both sexes during winter; NPQ values were lower in females than males. Furthermore, tocopherol levels were lower in females than in males, thus indicating that enhanced lipid peroxidation is associated with a lower photoprotection capacity in females. Indeed, α-tocopherol oxidation was higher in females than in males, thus suggesting a greater accumulation of ROS in the former. This is particularly interesting since it allows linking reduced photoprotection with increased lipid peroxidation in females. This is in agreement with previous studies suggesting that both LOX upregulation and downregulation of photoprotection mechanisms may be involved in the production of lipid-peroxide-derived signals (reviewed by Demmig-Adams *et al.*, 2013).

Females appeared to be able to reduce the extent of lipid peroxidation as time elapsed during the day. A decrease in MDA levels along the day was observed in females. MDA levels were higher in females than in males at midday, but the extent of lipid peroxidation was even higher at predawn. Furthermore, this occurred in parallel with an increased oxidation of α-tocopherol, and this antioxidant cannot be oxidized by singlet oxygen at predawn (in darkness). Higher lipoxygenase activity in females than in males (at predawn only) was the cause of higher MDA levels in the former. Furthermore, co-oxidation of fatty acids and α-tocopherol has been shown to occur *in vitro* (Hakansson and Jagerstad, 1990), which suggests that both the higher oxidation of α-tocopherol and increased lipid peroxidation in females compared to males at predawn was due to increased lipoxygenase activity. In turn, this was associated with reduced IPA levels in females compared to males at predawn. IPA is one of the most active cytokinins in *Pistacia lentiscus* leaves modulating leaf growth (Juvany *et al.*, 2013). Since cytokinin levels determine the capacity for cell division and the capacity of the organ to act as a sink for photoassimilates (reviewed by Roitsch and Ehness, 2000), results suggest that reductions in IPA in females at predawn may be associated with a sink limitation, which is, in turn, known to induce lipoxygenase activity (Fischer *et al.*, 1999). It appears therefore that increased lipid peroxidation in females compared to males at midday occurred irrespective of an increased lipoxygenase activity and was mainly due to reduced photoprotection; while a sink limitation at predawn resulted in increased lipoxygenase activity and accumulation of lipid-peroxide-derived signals. These results highlight the importance of considering the complexity of mechanisms leading to lipid peroxidation at the whole-plant level, as it has recently been pointed out for photoinhibition (Adams *et al.*, 2013). Furthermore, levels of other cytokinins and ABA were reduced at midday in females compared to males, which may be linked to a reduced vigour and defence capacity, which is in agreement with the parallel observation of reduced photoprotection capacity in females. It is still to be determined whether or not reduced ABA and cytokinin levels could transiently affect hydraulic conductivity or cell division, respectively, in females at midday, two aspects that warrant further investigations.

In conclusion, these results show that females are more sensitive to lipid peroxidation than males, but only during winter (when sex-related differences in reproductive effort over the year are the highest). Reduced photoprotection led to enhanced lipid peroxidation in females at midday, while increased lipoxygenase activity, probably mediated by a sink limitation-reduced cytokinin levels, was responsible for the increased lipid peroxidation at predawn. Finally, results showed that photoprotection capacity was higher in R relative to NR shoots in females, thus suggesting that females prioritized protection to fruit-bearing shoots.

## Supplementary material

Supplementary data are available at *JXB* online.

Supplementary Table S1. Climatological conditions at midday during Experiment 1

Supplementary Fig. S1. Seasonal variations in climatological conditions from March 2012 to February 2013

Supplementary Fig. S2. Sex-related differences in the seasonal variations in the relative leaf water content, maximum efficiency of PSII photochemistry, and levels of chlorophyll a+b and malondialdehyde in *Pistacia lentiscus*

Supplementary Fig. S3. Sex-related differences in the seasonal variations in levels of total anthocyanins, carotenoids, and α-tocopherol in *Pistacia lentiscus*

Supplementary Fig. S4. Sex-related differences during winter in the relative leaf water content of *Pistacia lentiscus*

Supplementary Fig. S5. Sex-related variations during winter in the levels of total anthocyanins, carotenoids, and α-tocopherol in *Pistacia lentiscus*

Supplementary Fig. S6. Sex-related differences in the diurnal variations in levels of total anthocyanins, carotenoids, and α-tocopherol in *Pistacia lentiscus* during winter

Supplementary Fig. S7. Sex-related differences in the diurnal variations in levels of total anthocyanins, carotenoids, and α-tocopherol in *Pistacia lentiscus* during winter (expressed per unit of chlorophyll)

Supplementary Fig. S8. Sex-related differences in the diurnal variations in levels of zeatin, zeatin riboside, and 2-isopentenyladenine in *Pistacia lentiscus* during winter

Supplementary Fig. S9. Sex-related differences during winter in the instantaneous water use efficiency response curves to photosynthetically active photon flux density in *Pistacia lentiscus*

Supplementary Data
