# Supplementary material for: Sex-related differences in lipid peroxidation and photoprotection in Pistacia lentiscus
Source: J Exp Bot. 2013 Dec 30;65(4):1039–49. doi: 10.1093/jxb/ert446 (PMC3935561; doi:10.1093/jxb/ert446)
Supplement: Supplementary Data [file supp_65_4_1039__index.html]

Sex-related differences in lipid peroxidation and photoprotection in Pistacia lentiscus — Sex-related differences in lipid peroxidation and photoprotection in Pistacia lentiscus — Supplementary Data 

# Sex-related differences in lipid peroxidation and photoprotection in *Pistacia lentiscus*

## Supplementary Data

Data files

**Files in this Data Supplement:**

- Supplementary Data - Supplementary Data
