# Supplementary material for: Sex-related differences in lipid peroxidation and photoprotection in Pistacia lentiscus
Source: J Exp Bot. 2013 Dec 30;65(4):1039–49. doi: 10.1093/jxb/ert446 (PMC3935561; doi:10.1093/jxb/ert446)

Supporting Information

Sex-related differences in lipid peroxidation and photoprotection in *Pistacia lentiscus*

Marta Juvany, Maren Müller, Marta Pintó-Marijuan, and Sergi Munné-Bosch

Table S1 Climatological conditions at midday (photosynthetically-active photon flux density [PPFD], air temperature [T_{air}] and relative humidity [RH]) during the measurements of Experiment 1.

Season	Date	PPFD (µmol m^{-2} s^{-1})	T_{air} (ºC)	RH (%)
Spring	28 March	1442	23.0	25
Summer	18 July	1940	32.0	38
Autumn	29 October	1152	14.5	36
Winter	24 January	876	10.0	52

Fig. S1 Seasonal variations in climatological conditions (monthly average air temperature [Tair], monthly average relative humidity and monthly precipitation) from March 2012 to February 2013.

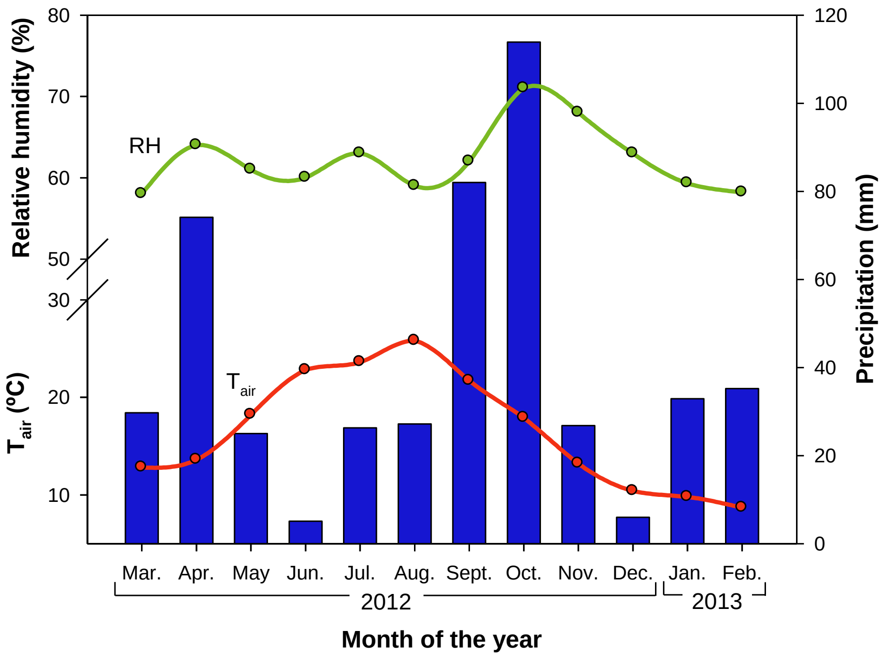

Fig. S2 Sex-related differences in the seasonal variations in the relative leaf water content (RWC), maximum efficiency of PSII photochemistry (F_v/F_m ratio), and levels of chlorophyll (Chl) a+b and malondialdehyde (MDA) in *P. lentiscus*. Data represent the mean ± SE of n=6 individuals. Significant differences between groups were tested by two-way factorial analyses of variance (ANOVA) with time and plant sex as factors. An asterisk indicates significant differences between males and females at a given time point (Student's t-test, P<0.05). NS, not significant.

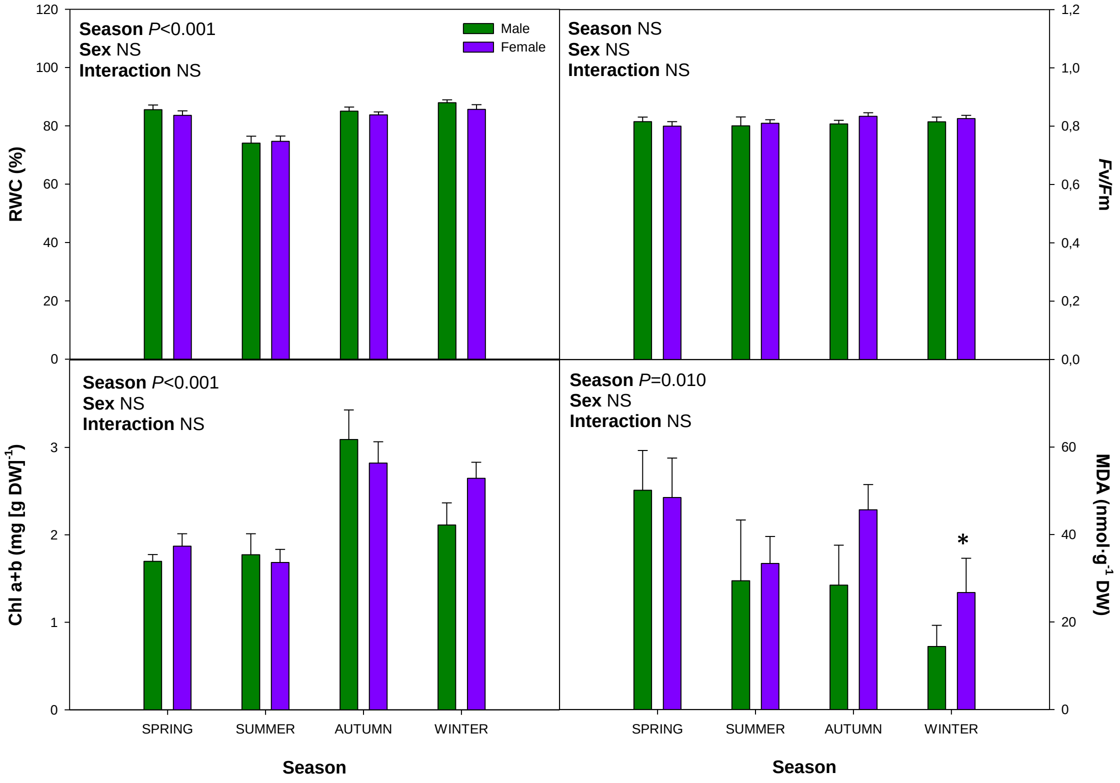

Fig. S3 Sex-related differences in the seasonal variations in levels of total anthocyanins (Ant), carotenoids (Car) and α-tocopherol (α-Toc), expressed per g of dry weight (DW) and per unit of chlorophyll (Chl) a+b in *P. lentiscus*. Data represent the mean ± SE of n=6 individuals. Significant differences between groups were tested by two-way factorial analyses of variance (ANOVA) with time and plant sex as factors. An asterisk indicates significant differences between males and females at a given time point (Student's t-test, $P<0.05$). NS, not significant.

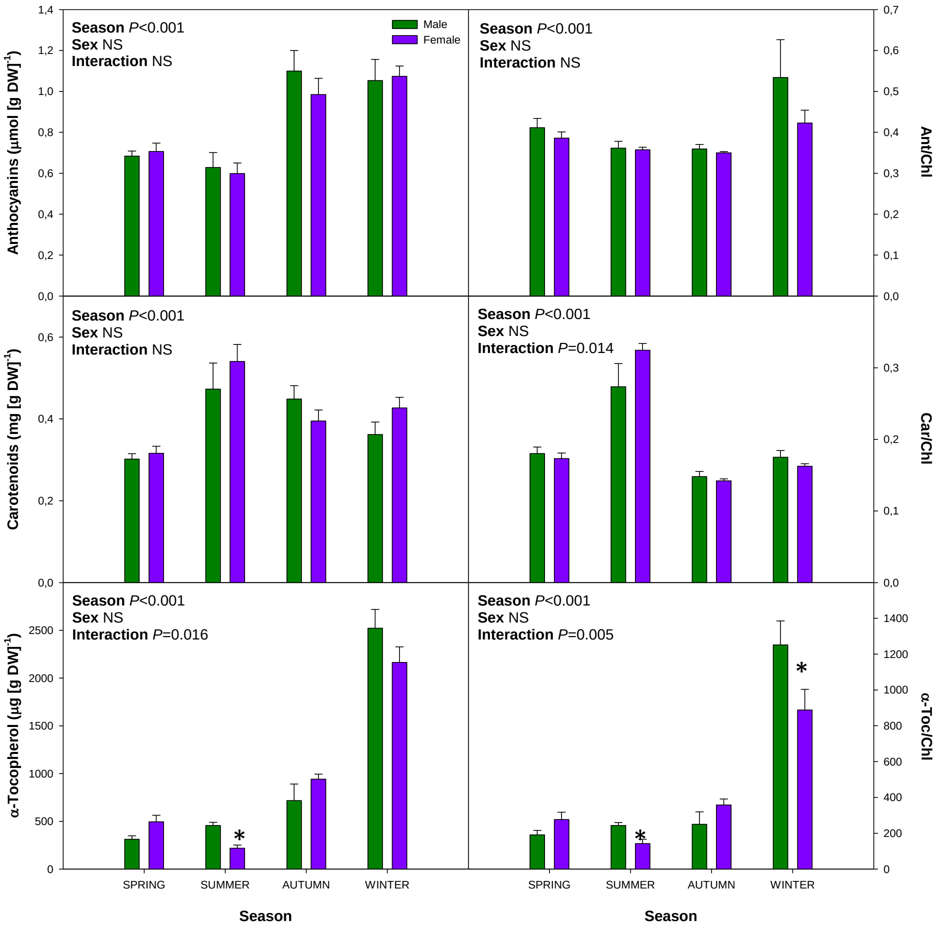

Fig. S4 Sex-related differences during winter in the relative leaf water content (RWC) of *P. lentiscus*. Data represent the mean ± SE of n=6 individuals. Significant differences between groups were tested by two-way factorial analyses of variance (ANOVA) with time and plant sex (females *vs.* males), shoot (reproductive -R- *vs.* non-reproductive – NR-) or fruit (shoots with and without fruits) as factors. NS, not significant.

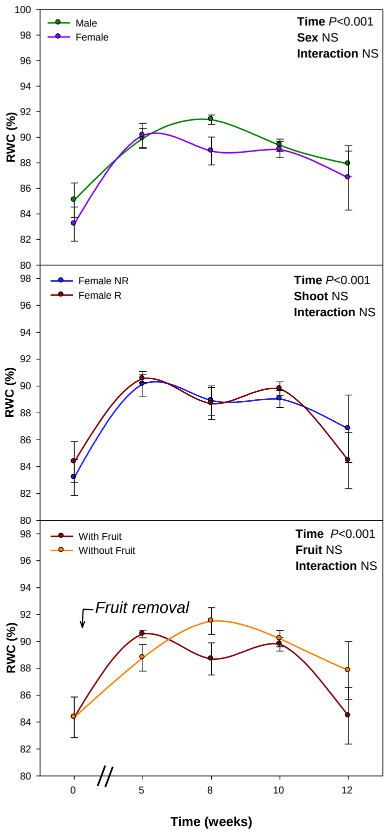

Fig. S5 Sex-related variations during winter in the levels of total anthocyanins (Ant), carotenoids (Car) and α-tocopherol (α-Toc),expressed per unit of chlorophyll (Chl) in *P. lentiscus*. Data represent the mean ± SE of n=6 individuals. Significant differences between groups were tested by two-way factorial analyses of variance (ANOVA) with time and plant sex (females *vs.* males), shoot (reproductive -R- *vs.* non-reproductive –NR-) or fruit (shoots with and without fruits) as factors. NS, not significant.

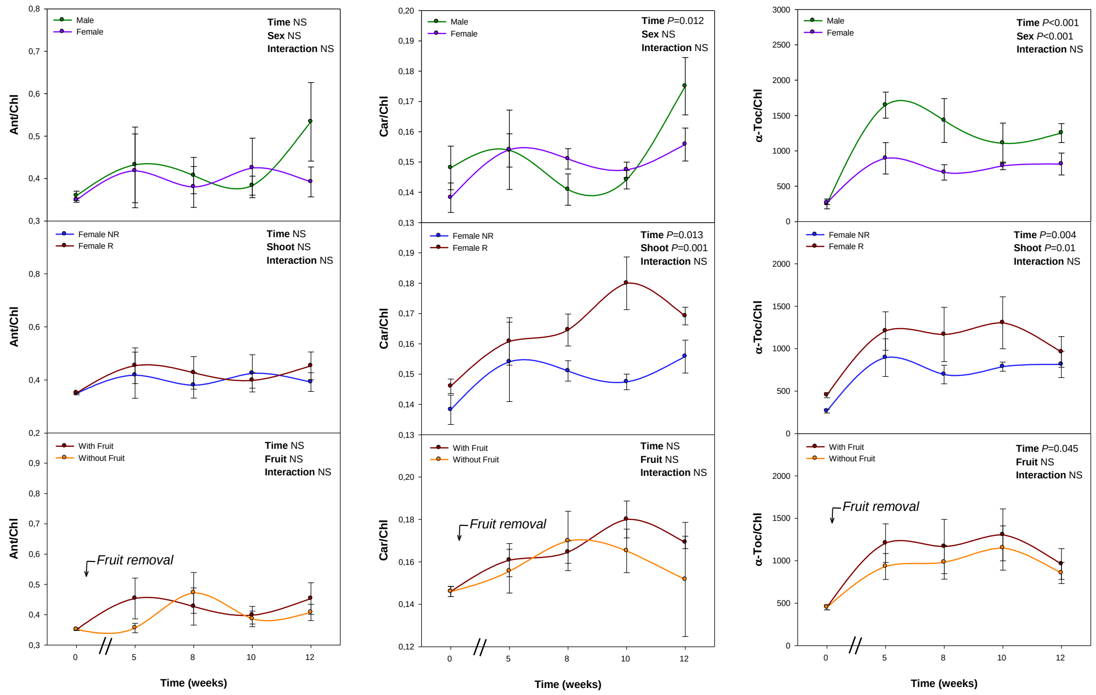

Fig. S6 Sex-related differences in the diurnal variations in levels of total anthocyanins (Ant), carotenoids (Car) and α-tocopherol (α-Toc) in *P. lentiscus* during winter (25 January). Data represent the mean ± SE of n=6 individuals. Significant differences between groups were tested by two-way factorial analyses of variance (ANOVA) with time and plant sex (females *vs.* males), shoot (reproductive -R- *vs.* non-reproductive –NR-) or fruit (shoots with and without fruits) as factors. An asterisk indicates significant differences between males and females, R and NR shoots, or shoots with and without fruits at a given time point (Student's t-test, *P*<0.05). NS, not significant.

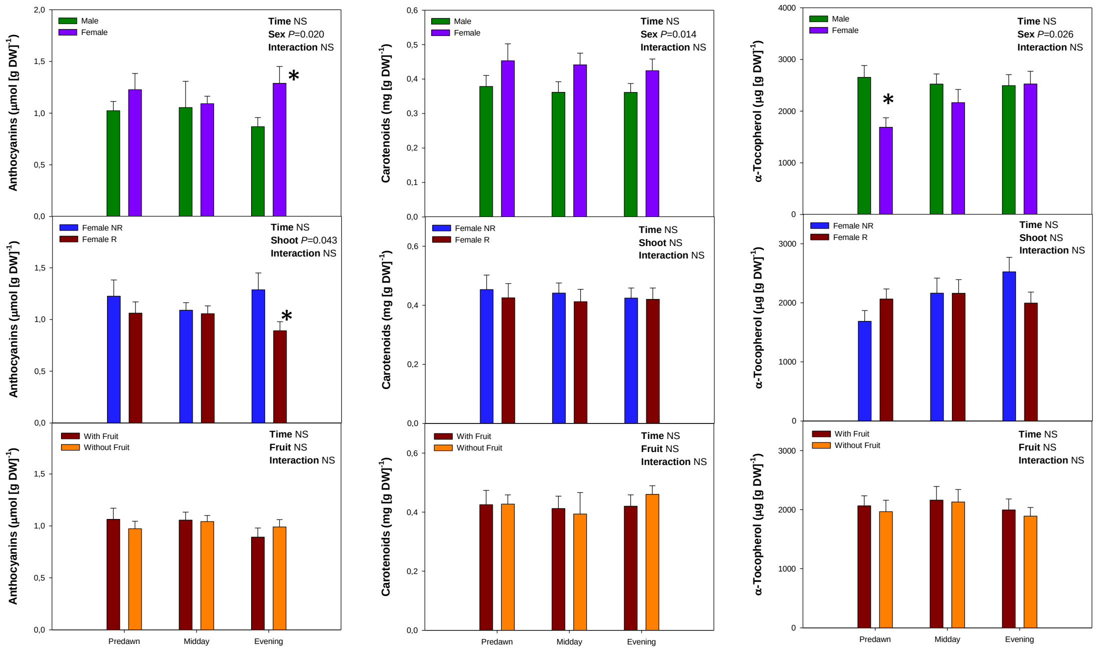

Fig. S7 Sex-related differences in the diurnal variations in levels of total anthocyanins (Ant), carotenoids (Car) and α-tocopherol (α-Toc) expressed per unit of chlorophyll (Chl) in *P. lentiscus* during winter (25 January). Data represent the mean ± SE of n=6 individuals. Significant differences between groups were tested by two-way factorial analyses of variance (ANOVA) with time and plant sex (females *vs.* males), shoot (reproductive -R- *vs.* non-reproductive –NR-) or fruit (shoots with or without fruits) as factors. An asterisk indicates significant differences between males and females, R and NR shoots, or shoots with and without fruits at a given time point (Student's t-test, *P*<0.05). NS, not significant.

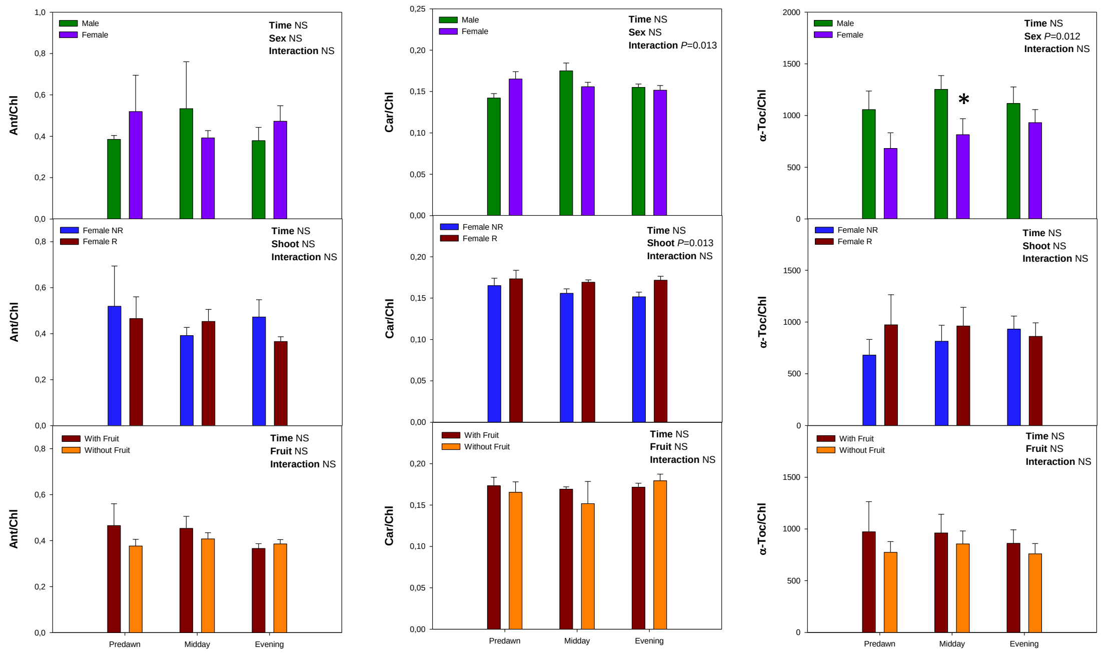

Fig. S8 Sex-related differences in the diurnal variations in levels zeatin (Z), zeatin riboside (ZR) and 2-isopentenyladenine (2iP) in *P. lentiscus* during winter (25 January). Data represent the mean ± SE of n=6 individuals. Significant differences between groups were tested by two-way factorial analyses of variance (ANOVA) with time and plant sex (females *vs.* males), shoot (reproductive -R- *vs.* non-reproductive –NR-) or fruit (shoots with or without fruits) as factors. An asterisk indicates significant differences between males and females at a given time point (Student's t-test, *P*<0.05). NS, not significant.

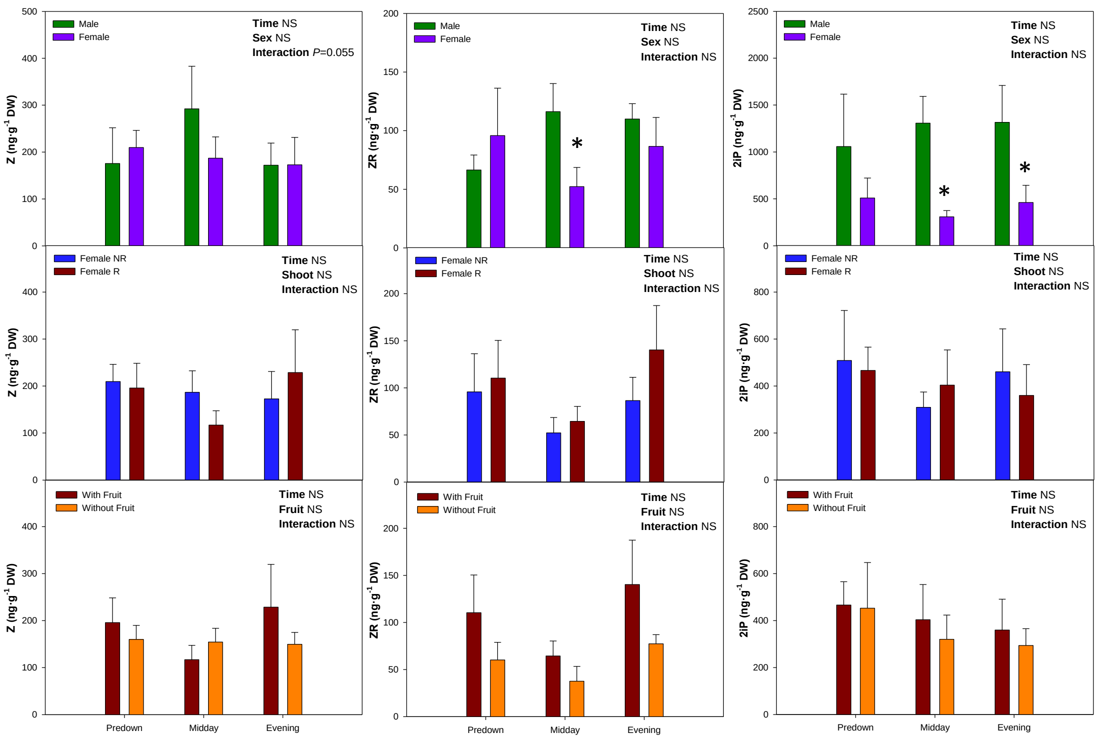

Fig. S9 Sex-related differences during winter in the instantaneous water use efficiency (WUE) response curves to photosynthetically-active photon flux density (PPFD) in *P. lentiscus*. Data represent the mean ± SE of n=6 individuals. Significant differences between groups were tested by two-way factorial analyses of variance (ANOVA) with time and plant sex (females *vs.* males), shoot (reproductive -R- *vs.* non-reproductive –NR-) or fruit (shoots with and without fruits) as factors. NS, not significant. Green shading indicates midday sampling PPFD range during winter.

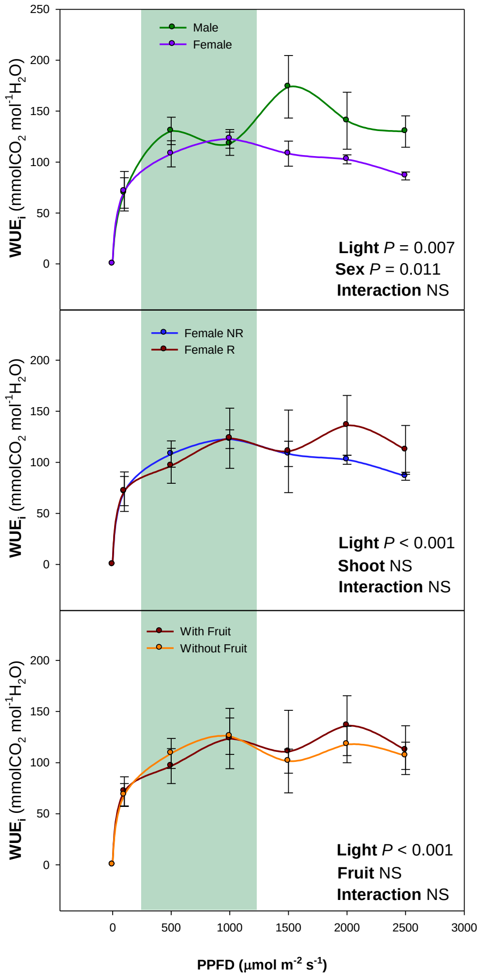

Supplement: Supplementary Data [file supp_ert446_jexbot112524_file001.pdf]
